# Bio-organic fertilizer affects secondary cell wall biosynthesis of *Dendrocalamus farinosus* by inhibiting the phenylpropanoid metabolic pathway

**DOI:** 10.1186/s12870-024-05825-8

**Published:** 2024-11-22

**Authors:** Shangmeng Li, Ying Cao, Boya Wang, Wei Fan, Shanglian Hu

**Affiliations:** 1 Bamboo Research Institute, School of Life Science and Engineering, Southwest University of Science and Technology, Mianyang, China; 2 Sichuan Provincial Forestry and Grass Land Key Laboratory for Conservation and Sustainable Utilization of Bamboo Genetic Resources in Southwest of China, Mianyang, China

**Keywords:** *Dendrocalamus farinosus*, Organic and microbial fertilizers, Fertilization, Transcriptome, Metabolome, Secondary cell wall

## Abstract

Bamboo, as a timber plant, holds significant environmental and economic value. *Dendrocalamus farinosus* is particularly valuable as it serves both as a source of bamboo shoots and timber, offering high yield, strong disease resistance, and superior fiber quality. Our previous study demonstrated that bio-organic fertilizers promoted the growth of *D. farinosus* and significantly altered the cellulose and lignin content, key components of the secondary cell wall in culms. However, the underlying regulatory mechanisms remain unclear. In this study, we used metabolomic and transcriptomic analyses to uncover the potential mechanisms by which bio-organic fertilizers affect the secondary cell wall biosynthesis in *D. farinosus*. A total of 1,437 metabolites were identified, with 20 differential metabolites significantly enriched in the phenylpropanoid metabolic pathway in bamboo shoots (7 upregulated; 13 downregulated). We identified 8,075 differentially expressed genes in bamboo shoots, including 72 genes potentially involved in lignin and flavonoid biosynthesis (6 upregulated; 66 downregulated). In internodes, we identified 5,324 differentially expressed genes, including 83 genes potentially involved in secondary cell wall biosynthesis (43 upregulated; 39 downregulated). Quantitative real-time PCR (qRT-PCR) validated the expression patterns of 8 key genes in internodes. The results suggest that bio-organic fertilizers may affect secondary cell wall biosynthesis in internodes by inhibiting the phenylpropanoid metabolic pathway in *D. farinosus* shoots. Our study offers insights into the efficient utilization of bamboo and lignocellulosic biomass, serving as a valuable resource for future research.

**Supplementary Information:**

The online version contains supplementary material available at 10.1186/s12870-024-05825-8.

## Introduction

With rapid global economic growth, the environmental degradation and resource demands driven by industrialization and urbanization are intensifying. The growing demand for construction materials, paper, and sustainable solutions has fueled research into plant biomass. As forest resources dwindle, developing and utilizing non-wood biomass has become essential. In this context, bamboo stands out as a promising feedstock due to its short growth cycle. It also serves as a strong alternative to wood, offering excellent mechanical strength, tensile resistance, and high elasticity [1]. This superior performance culms from the supramolecular network of complex carbohydrates and aromatic polymers in the secondary cell wall (SCW) [2].

The SCW is essential for plant strength, durability, and overall function [3]. Unlike the primary cell wall, which forms during early growth, the SCW is deposited after cell expansion ceases, providing increased structural support [4]. This process is particularly important for cells responsible for mechanical strength, water transport, and long-term structural integrity, such as xylem, fibers, and sclerenchyma cells [5]. The biosynthesis of the SCW is a tightly regulated process that involves the deposition of key components, including lignin, cellulose, and hemicellulose [6].

Phenylpropanoid metabolism is essential in plants and plays a central role in lignin biosynthesis, a key component of the SCW. The biosynthesis and diversification of phenylpropanoid metabolites are facilitated by a coordinated cascade of enzymes, including oxygenases, oxidoreductases, ligases, and polytransferases, which chemically modify the metabolite backbone through acylation, methylation, glycosylation, and hydroxylation [7, 8]. In switchgrass, a primary lignocellulosic feedstock for renewable bioenergy, alterations in SCW structure have been achieved through the regulation of transcription factors. For instance, RNA interference (RNAi) has been used to downregulate caffeic acid O-methyltransferase (*COMT*), 4-coumarate A ligase (*4CL*), and cinnamyl alcohol dehydrogenase (*CAD*), impacting SCW biosynthesis [9, 10]. Additionally, tissue-specific expression of v-myb avian myeloblastosis viral oncogene homolog (*MYB4*) in green tissues and downregulation of the pectin biosynthesis gene *GAUT4* have been reported [11, 12]. Overexpression of caffeoyl-coenzyme A-O-methyltransferase (*DfCCoAOMT*) in *D. farinosus* significantly increased lignin content and xylem thickness in transgenic plant, as demonstrated by Wei [13]. Lignin content plays a critical role in determining cell wall recalcitrance. In *Arabidopsis thaliana*, disruption of MYB transcription factors such as *MYB20*, *MYB42*, *MYB43*, and *MYB85* resulted in developmental defects and a marked reduction in lignin biosynthesis [14]. Hybridization of COMT-downregulated transgenic switchgrass with field-selected varieties enhanced biomass traits, offering new insights into modifying cell wall recalcitrance through genetic breeding [15]. Furthermore, the development of low-recalcitrant varieties has been explored by assessing natural populations of *Salix viminalis* for heritable traits, lignin S/G ratio, and shoot diameter [16].

Despite substantial understanding of general SCW biosynthesis mechanisms in plants, there remain significant gaps in knowledge regarding how environmental factors, such as fertilizers, influence this process in bamboo. Specifically, there is a lack of in-depth research on how bio-organic fertilizers affect the phenylpropanoid pathway in bamboo, particularly at different developmental stages (bamboo shoots vs. bamboo culms).

Previous studies have demonstrated that exogenous indole acetic acid (IAA) promotes internode elongation and increases plant height in moso bamboo [17], while treatments with exogenous sucrose and gibberellin (GA) resulted in significantly longer internodes compared to controls [18]. Our previous studies showed that exogenous bio-organic fertilizer OFBa significantly affected the growth and development of *D. farinosus*, especially affecting cellulose and lignin content [20]. *D. farinosus* is a promising non-timber biomass plant, offering high disease resistance, excellent fiber quality, and favorable economic traits [19]. Thus, understanding the biosynthesis and regulation of the secondary cell wall (SCW) in *D. farinosu* under bio-organic fertilizer treatments is crucial for the effective utilization of bamboo wood and lignocellulosic biomass.

Although several methods have been employed to study genetic regulations related to SCW synthesis, RNA-seq based on next-generation sequencing has become a routine approach for bamboo research [21–24]. With the advancement of metabolomics technology and its integration with transcriptome research in bamboo shoots, phenotypic characterization using these methods has been widely validated. For instance, the regulation and biosynthesis of bitter substances have been analyzed in *Pleioblastus amarus*, *Bambusa oldhamii*, and *Dendrocalamopsis oldhamii* [25–27]. Despite these advancements, little has been reported on metabolism and transcriptional regulation during SCW biosynthesis in *D. farinosus*, especially under bio-organic fertilizer induction. This study utilized transcriptomics and metabolomics techniques to investigate the effects of bio-organic fertilizer (organic fertilizer plus *Bacillus amyloliquefaciens*) on SCW biosynthesis in potted *D. farinosus*, focusing on the transition from bamboo shoots to culms. Our primary goal was to elucidate the regulatory mechanisms by which bio-organic fertilizers affect SCW formation in *D. farinosus*, laying the foundation for the efficient utilization of bamboo wood and lignocellulosic biomass.

## Results

### Effects of OFBa on bamboo shoots metabolic profiling

**Fig. 1:**
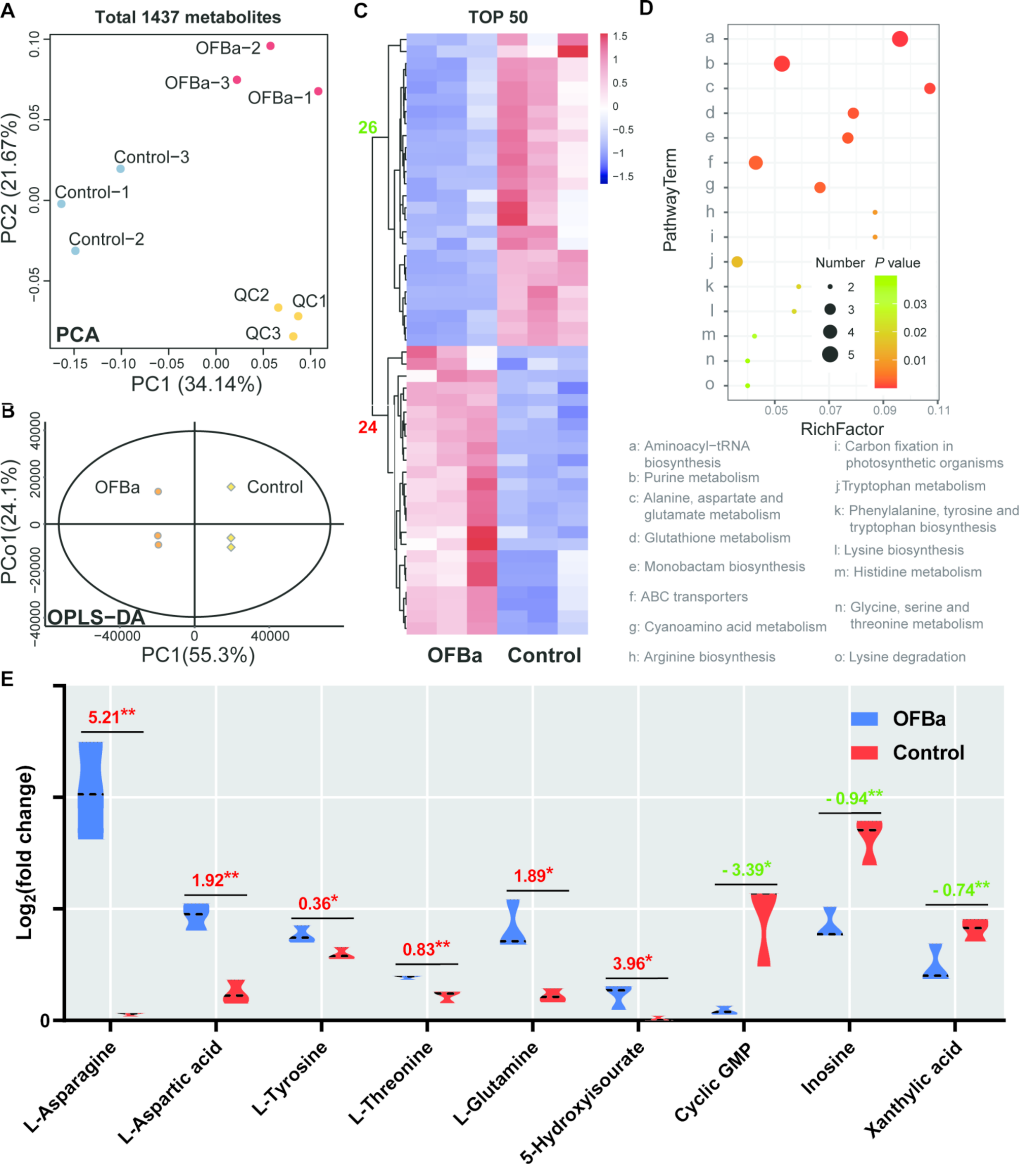
Metabolism profile of *Dendrocalamus farinosus* shoots under OFBa treatment. Principle component analysis (PCA) (**A**), orthogonal partial least squares discriminant analysis (OPLS-DA) (**B**), heat map of top 50 metabolisms (**C**), enrichment pathway analysis (**D**) and top ten metabolites for the first three enrichment pathways (**E**). Note: In Fig. 1A, QC represents quality control samples. In Fig. 1E, the data represents the ratio of the average expression of metabolites in the two groups of samples (Log_2_FC), with positive value indicating up-regulation and negative value indicating down-regulation. Tukey post-hoc tests were used to calculate the differences between the control and OFBa. * indicates significant difference at 0.05 level. ** indicates significant difference at 0.01 level (*n* = 3). Detailed annotation information of all metabolites was shown in Table S1. Detailed annotation information of differentially expressed metabolites was shown in Table S2. Detailed data for all soil metabolites analyzed was shown in Table S3. OFBa: bio-organic fertilizer (containing *Bacillus amyloliquefaciens*)

To investigate the overall metabolic changes in bamboo shoots treated with OFBa, we conducted metabolic profiling. Figure S1 presents the phenotypes of bamboo shoots used for metabolomic and transcriptomic analyses. A total of 1,437 metabolites were identified through non-targeted liquid chromatography-mass spectrometry (LC-MS) in the metabolomics analysis. To examine relationships between metabolites, Principal Component Analysis (PCA) and Orthogonal Partial Least Squares-Discriminant Analysis (OPLS-DA) were applied to identify differential metabolites between two sample groups (Control and OFBa). The PCA load diagram showed significant separation in the first and second principal components of the bamboo shoots’ metabolic profiles after OFBa treatment, explaining 34.14% and 21.67% of the variance, respectively (Fig. 1A). OFBa treatment alters the metabolic processes in bamboo shoot. The 200 response permutation testing (RPT) of the OPLS-DA model indicated minimal overfitting. The OPLS-DA model parameters showed predictive rate R^2^Y(cum) = 1 and explanatory rate Q^2^ (cum) = 0.98, suggesting that the model effectively explains and predicts the differences between the two sample groups (Fig. S2A). OPLS-DA revealed a clear separation in the first principal component of bamboo shoot metabolites following OFBa treatment, explaining 55.3% of the data variation (Fig. 1B). Based on Variable Importance in Projection (VIP > 1, *P* < 0.05) in the OPLS-DA model, 116 differentially expressed metabolites (DEMs) were identified, including 52 upregulated and 64 downregulated metabolites (Fig. S2B). The heatmap displays the top 50 differentially expressed metabolites, consisting of 24 upregulated and 26 downregulated metabolites (Fig. 1C, Table S2). The top four enrichment groups of DEMs in bamboo shoots treated with OFBa were organic acids and derivatives (24 in total), phenylpropanoids and polyketides (20 in total), lipids and lipid-like molecules (19 in total), and organic oxygen compounds (16 in total) (Fig. S2C). Mapping the DEMs to the Kyoto Encyclopedia of Genes and Genomes (KEGG) pathway database revealed significant enrichment in pathways related to aminoacyl-tRNA biosynthesis, purine metabolism, and alanine, aspartate, and glutamate metabolism (Fig. 1D, Table S4). In the top three enriched pathways, levels of l-asparagine (5.21-fold), l-aspartic acid (1.92-fold), l-tyrosine (0.36-fold), l-threonine (0.83-fold), l-glutamine (1.89-fold), and 5-hydroxyisouric acid (3.96-fold) were significantly elevated in the OFBa treatment compared to the control (*P* < 0.05). Conversely, cyclic GMP (3.39-fold), inosine (0.94-fold), and xanthylic acid (0.74-fold) were significantly reduced under OFBa treatment relative to the control (*P* < 0.05) (Fig. 1E).

## Bamboo shoots grown under OFBa exhibited a downregulated of phenylpropanoid metabolic

**Fig. 2:**
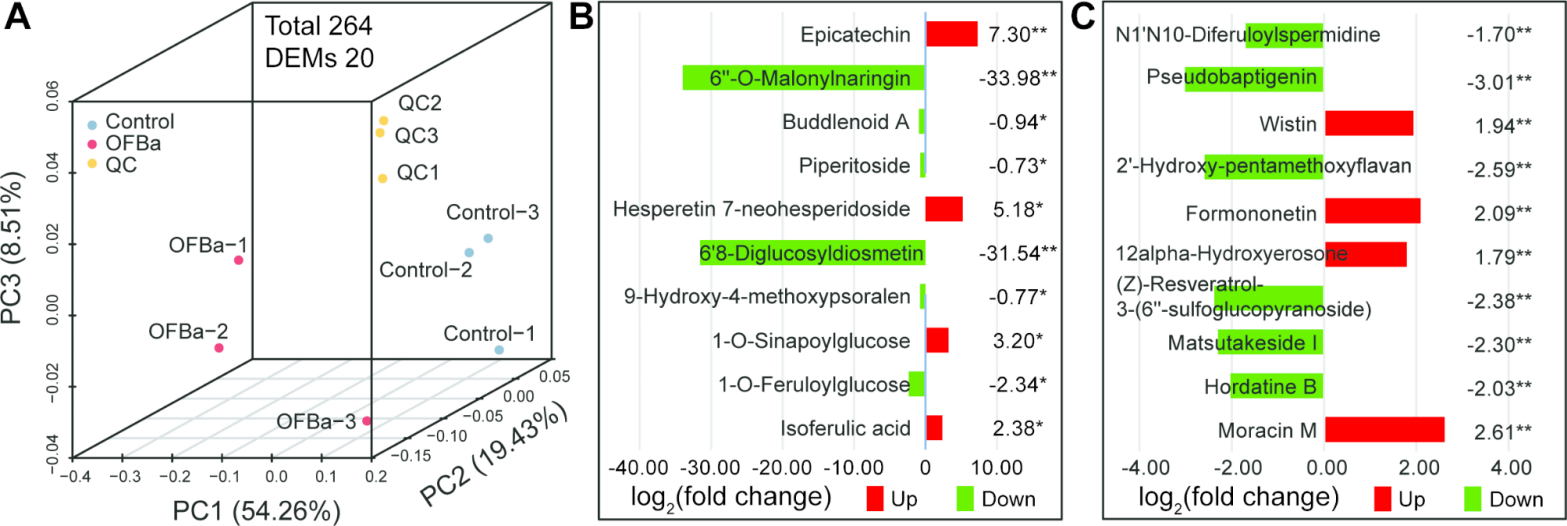
Principal component analysis (PCA) on the effects of OFBa on the expression pattern of metabolites in phenylpropanoids metabolism pathway in *Dendrocalamus farinosus* shoots. Note: In Fig. 2B and C, the data represents the ratio of the average expression of metabolites in the two groups of samples (log_2_FC), with positive value indicating up-regulation and negative value indicating down-regulation. * indicates significant difference at 0.05 level. ** indicates significant difference at 0.01 level. OFBa: bio-organic fertilizer (containing *Bacillus amyloliquefaciens*)

To investigate the accumulation of metabolites in the ‘phenylpropanoids and polyketides’ enrichment group, we detected 264 metabolites associated with phenylpropanoid metabolism, identifying 20 differentially expressed metabolites (DEMs) (VIP > 1, *P* < 0.05). PCA loading plots indicated that the phenylpropanoid metabolic pathway in bamboo shoots may have been significantly influenced by OFBa (Fig. 2A). Of the identified metabolites, 13 were downregulated, and 7 were upregulated. The results revealed a significant inhibition in the expression of 6’’-O-malonylnaringin (downregulated: − 33.98, *P* < 0.01), 6’8-diglucosyldiosmetin (downregulated: − 31.54, *P* < 0.01), and pseudobaptigenin (downregulated: − 3.01, *P* < 0.01) within 90 days of OFBa treatment. Meanwhile, the production of epicatechin, hesperetin 7-neohesperidoside, and 1-O-sinapoylglucose increased by 7.30, 5.18, and 3.20 times, respectively (Fig. 2B and C). These findings suggest that OFBa induced an imbalance in plant phenylpropanoid metabolites.

## Bamboo shoots grown under OFBa showed a significantly downregulated of cellular and primary metabolic

**Fig. 3:**
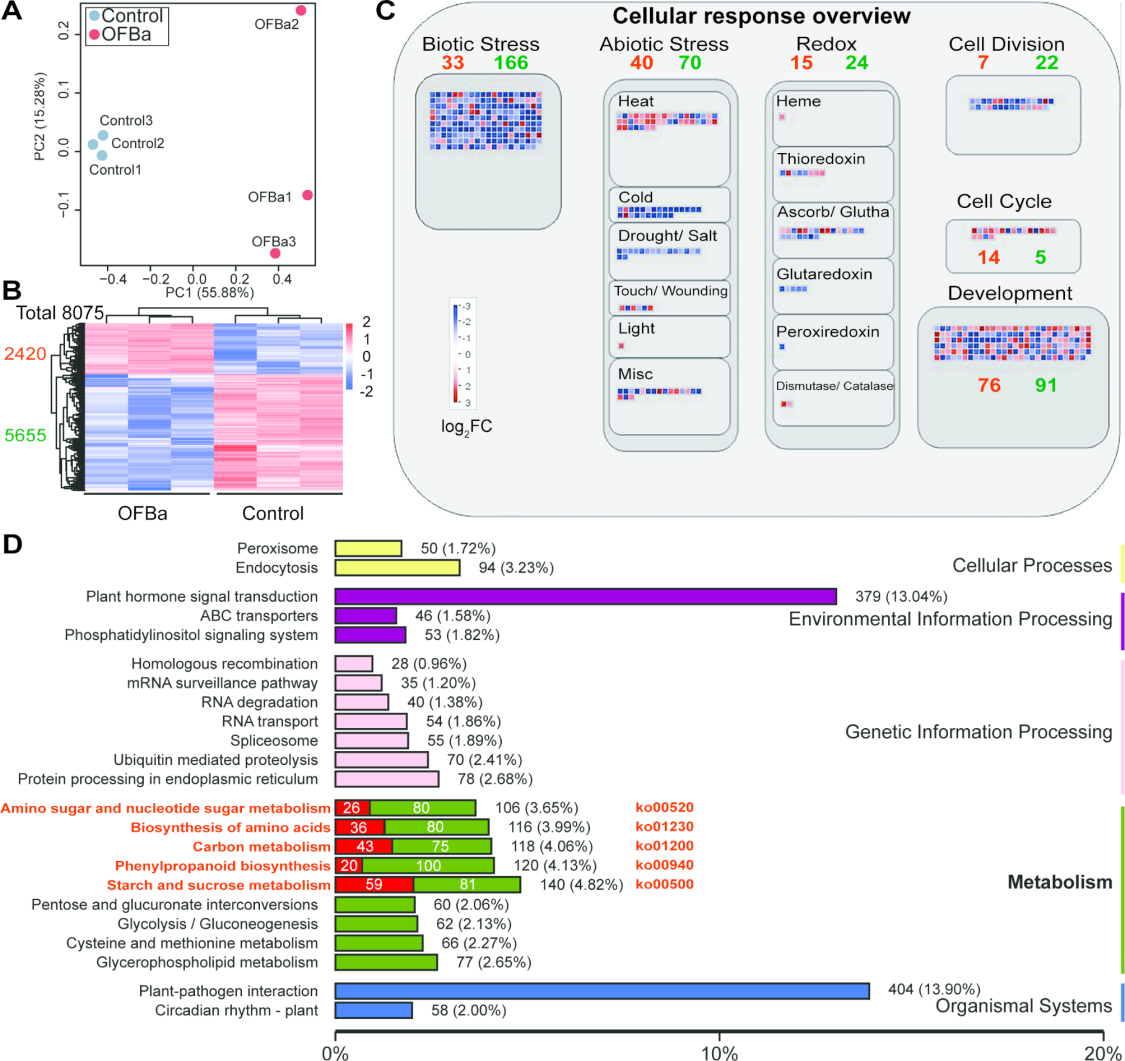
Transcriptome analysis of *Dendrocalamus farinosus* shoots under OFBa. Principle component analysis (PCA) (**A**), heat map analysis (**B**), visual analysis (**C**) and Kyoto Encyclopedia of Genes and Genomes (KEGG) enrichment pathway analysis (**D**) of differentially expressed genes (DEGs). Note: Visual analysis was completed by using Mapman (3.6.1RC1). OFBa: bio-organic fertilizer (containing *Bacillus amyloliquefaciens*). In Fig. 3D (Metabolism), red font indicates the number of immediate homologs (ko) associated with each metabolic pathway. Red boxes and the numbers within them denote the upregulated metabolites and their counts, while green boxes and their numbers represent the downregulated metabolites and their counts. Percentages indicate the proportion of DEGs in each metabolic pathway relative to the total number of DEGs. OFBa: bio-organic fertilizer (containing *Bacillus amyloliquefaciens*)

To elucidate the molecular basis underlying the inhibition of phenylpropanoid metabolism by OFBa treatment, we performed global transcriptomic analysis using RNA-seq. The robustness and reliability of the RNA-seq data are demonstrated by the PCA plot, which shows clear distinction between the control and OFBa-treated groups (Fig. 3A). The correlation plot reveals high consistency in gene expression levels across replicates (Fig. S3). Differential gene expression analysis identified 8,075 differentially expressed genes (DEGs) (Fig. 3B, Table S5), including 2,420 significantly upregulated and 5,655 significantly downregulated (log2FC [fold change] > 1.5, *P* < 0.05). Visual analysis revealed significant alterations in genes related to biotic/abiotic stress responses, redox processes, cell division, cell cycle, and development in OFBa-treated bamboo shoot cells (Fig. 3C). Bamboo shoots grown under OFBa treatment showed significant downregulation of genes associated with biotic/abiotic stress responses (73 upregulated; 236 downregulated), cell division (15 upregulated; 24 downregulated), and cell wall organization (7 upregulated; 22 downregulated). In contrast, genes related to the cell cycle exhibited significant upregulation (14 upregulated; 5 downregulated) (Fig. 3C, Table S6). Significant changes were observed in KEGG-enriched pathways, including starch and sucrose metabolism (ko00500), phenylpropanoid metabolism (ko00940), carbon metabolism (ko01200), amino acid biosynthesis (ko01230), and amino sugar and nucleotide sugar metabolism (ko00520). Specifically, OFBa influenced 140 genes (59 upregulated; 81 downregulated) in starch and sucrose metabolism, 120 genes (20 upregulated; 100 downregulated) in phenylpropanoid metabolism, 118 genes (43 upregulated; 75 downregulated) in carbon metabolism, 116 genes (36 upregulated; 80 downregulated) in amino acid biosynthesis, and 106 genes (26 upregulated; 80 downregulated) in amino sugar and nucleotide sugar metabolism (Fig. 3D).

## Regulation mechanism of OFBa on phenylpropanoid metabolic pathway of bamboo shoots

**Fig. 4:**
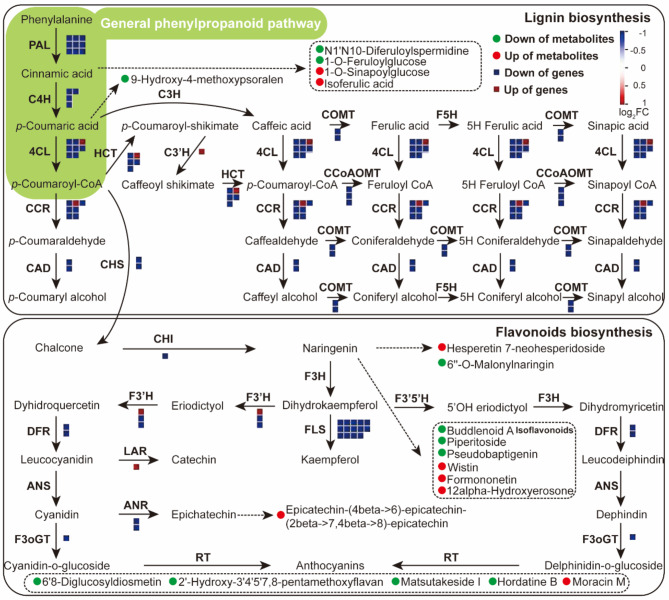
Expression patterns of key genes and metabolites related to the phenylpropanoid pathway in *Dendrocalamus farinosus* shoots under OFBa. Blue and red squares represent down and upregulated DEGs, and green and red circles represent down and upregulated differentially expressed metabolites (DEMs), respectively. Solid and dashed arrows represent direct and indirect effects, respectively. Metabolic pathway adapted from Mapman (3.6.0RC1) and amendments involved displaying the newly identified genes and metabolites under this study and the Kyoto Encyclopedia of Genes and Genomes (KEGG) portal used for analysis. OFBa: bio-organic fertilizer (containing *Bacillus amyloliquefaciens*)

The phenylpropanoid metabolic pathway is a critical metabolic process in plants, and genes encoding key enzymes are vital for its regulation and study [38]. In the phenylpropanoid pathway, bamboo shoots grown under OFBa showed significant down-regulation in the expression of lignin and flavonoid biosynthesis genes, with 6 genes upregulated and 66 downregulated (Fig. 4, Table S7). OFBa treatment downregulated 9 phenylalanine ammonia-lyase 1 (*PAL1*), 9 cinnamate 4-hydroxylases (*C4H*), 7 4-coumarate A ligases (*4CL*), 2 caffeic acid O-methyltransferases (*COMT*), 4 hydroxycinnamoyl transferases (*HCT*), 3 caffeoyl coenzyme A 3-O-methyltransferases (*CCoAOMT*), 6 cinnamoyl coenzyme A reductases (*CCR*), 2 cinnamyl alcohol dehydrogenases (*CAD*), 2 chalcone synthases (*CHS*), 1 chalcone isomerase (*CHI*), 13 flavonol synthases (*FLS*), 2 flavanone 3-hydroxylases (*F3’H*), 2 dihydroflavanol-4-reductases (*DFR*), 1 UDP-glucose: anthocyanidin 3-O-glucosyltransferase (*UFGT*), and 2 anthocyanidin reductases (*ANR*) compared to the control (Fig. 4, Table S7). Conversely, 1 *4CL*, 1 *HCT*, 1 *C3H*, 1 *CCR*, 1 *F3’H*, and 1 leucoanthocyanidin reductase (*LAR*) were upregulated in OFBa compared to the control (Fig. 4, Table S7). Additionally, OFBa significantly downregulated the metabolites including 6’’-O-malonylnaringin, matsutakeside I, buddlenoid A, 1-O-feruloylglucose, piperitoside, 2’-Hydroxy-3’,4’,5’,7,8-pentamethoxyflavan, hordatine B, pseudobaptigenin, N1,N10-diferuloylspermidine, and 6,8-diglucosyldiosmetin. OFBa significantly upregulated the metabolites including hesperetin 7-neohesperidoside, isoferulic acid, formononetin, 1-O-sinapoylglucose, moracin M, wistin, and 12alpha-hydroxyerosone (Fig. 4, Table S7).

## Cellular metabolic response of bamboo culms to OFBa treatment

**Fig. 5:**
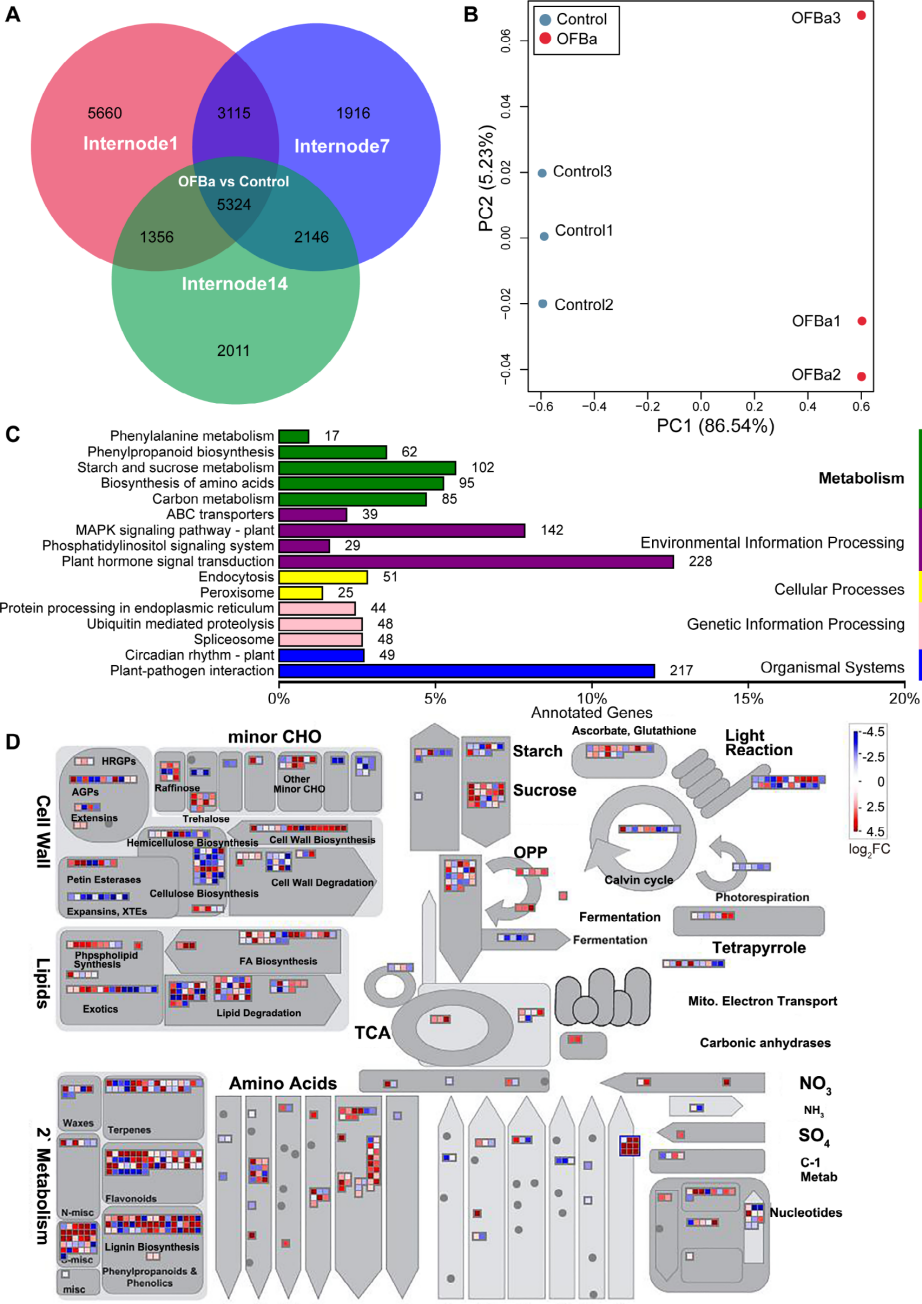
Transcriptome analysis of *Dendrocalamus farinosus* culms under OFBa treatment. Venn plots between internodes 1st, 7th, and 14th (**A**), principal component analysis (**B**), Kyoto Encyclopedia of Genes and Genomes (KEGG) enrichment pathway analysis (**C**) and visual analysis (**D**) of differentially expressed genes (DEG). Note: In Fig. 5A, Internode 1, internode 7 and internode 14 represent DEGs in the first internode, the seventh internode and the fourteenth internode, respectively, under OFBa compared to the control. OFBa represents DEGs shared among the three internodes. Mapman (3.6.1RC1) was used to visual analysis. OFBa: bio-organic fertilizer (containing *Bacillus amyloliquefaciens*)

Phloroglucinol staining of the bamboo culm revealed fewer vascular bundles and altered lignin content in OFBa-treated sections compared to the control (Fig. S4A). Our previous study showed that OFBa significantly reduced lignin content and increased cellulose content in the internode (Fig. S4B, C) [19]. To identify key genes regulating secondary cell wall (SCW) growth and development in *D. farinosus* under OFBa, we further analyzed the internode transcriptome data. RNA-seq analysis identified and annotated 75,861 genes (Table S8). Differential expression analysis revealed 15,455 DEGs in 1st internode (Internode1), 12,501 in 7th internode (Internode7), and 10,837 in 14th internode (Internode14) of *D. farinosus* under OFBa treatment compared to the control (Table S9). A Venn diagram of DEGs across internodes under OFBa treatment revealed 5,324 common DEGs (log2FC > 1, *P* < 0.05) (Fig. 5A, Table S9). We used these common DEGs for all subsequent analyses. Differential expression analysis identified 2,543 significantly downregulated DEGs and 2,781 significantly upregulated DEGs, as shown in the volcano plot (Fig. S5). Principal component analysis (PCA) showed distinct clustering between OFBa and control samples, indicating significant transcriptome differences between groups (Fig. 5B). KEGG enrichment analysis indicated that DEGs were primarily enriched in plant hormone signal transduction, plant-pathogen interactions, and primary metabolic pathways. *D. farinosus* grown under OFBa showed a signifcant variance of 102 genes in starch and sucrose metabolism, 95 in amino acid biosynthesis, 85 in carbon metabolism, 62 in phenylpropanoid metabolism, and 17 in phenylalanine metabolism (Fig. 5C). Visual analysis revealed a high enrichment of genes involved in cell wall biosynthesis (e.g., cellulose and hemicellulose biosynthesis) and secondary metabolites (e.g., lignin biosynthesis and flavonoids), both closely associated with SCW biosynthesis (Fig. 5D).

### OFBa broadly upregulate the expression of genes related to cellulose and xylan biosynthesis

**Fig. 6:**
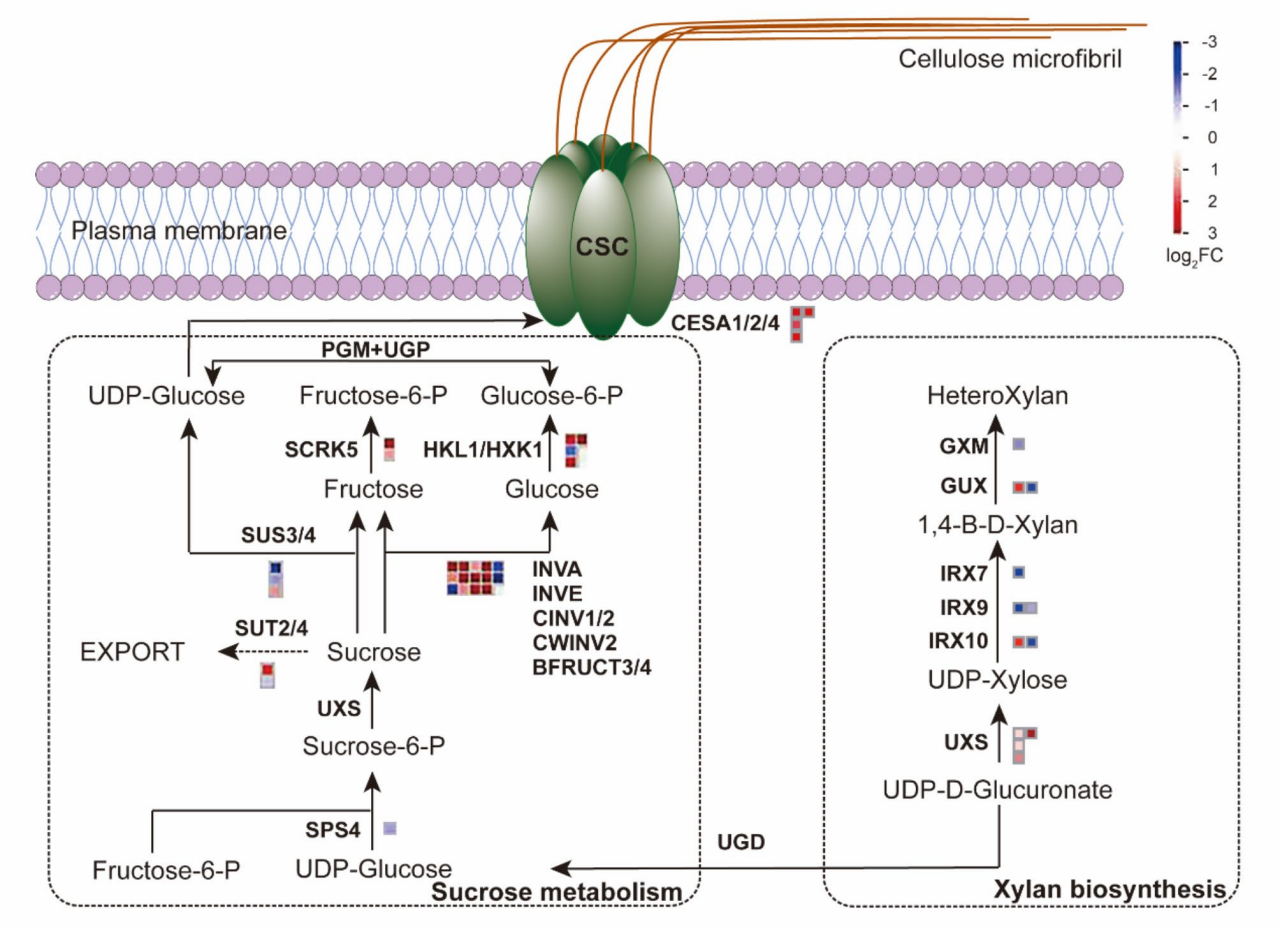
Expression patterns of genes related to cellulose and xylan biosynthesis in secondary cell walls of *Dendrocalamus farinosus* culms under OFBa. Blue and red squares represent down and upregulated DEGs, respectively. OFBa: bio-organic fertilizer (containing *Bacillus amyloliquefaciens*)

To further investigate the effect of OFBa on SCW biosynthesis, we analyzed the DEGs associated with the three primary components of the secondary wall: cellulose, hemicellulose, and lignin. Cellulose synthase and cellulose synthase-like (*CESA* and *CSL*) genes belong to the glycosyltransferase (GT) family 2 and are primarily involved in the biosynthesis of cellulosic and hemicellulosic components in plants [34–36]. A total of 50 *CESA* and *CSL* gene members, along with several structural proteins, were differentially expressed under OFBa, with 25 being upregulated and 25 downregulated (Fig. 6 and Table S10). Among these, 4 members homologous to *Arabidopsis thaliana CESA* (*CESA1*, *CESA2*, *CESA4*) were upregulated in OFBa compared to the control (Fig. 6 and Table S10). These findings suggest that these members may be involved in the biosynthesis of cellulose and hemicellulose polysaccharides in secondary and primary walls, respectively, under the influence of OFBa. The *CSL* genes were classified into eight distinct subgroups (*CSLA*-*CSLG*) [145]. 21 *CSL* gene members were expressed in the analyzed internodes. Compared to the control, 4 *CSLA09* genes and 1 *CSLD03* gene were upregulated in OFBa (Table S10). COBRA family genes encode glycosylphosphatidylinositol (GPI)-anchoring proteins involved in the assembly of crystalline cellulose during secondary cell wall formation. 2 *COBL4* genes were upregulated, while one COB gene was downregulated in OFBa compared to the control (Fig. 6 and Table S10). The extension of the xylan backbone requires irregular xylem 9 (*IRX9*) from the *GT43* family, xylan glucuronosyltransferase (*GUX*) from the *GT8* family, *IRX10* from the *GT47* family, and the putative homologous genes UDP-glucuronic acid decarboxylase (*UXS*) and glucuronoxylan 4-O-methyltransferase (GXM). Meanwhile, *IRX7* from the *GT47* family is involved in the synthesis of tetrasaccharides at the reducing end of the xylan. Compared to the control, 1 *IRX7*, 1 *IRX9*, and 1 *IRX10* were downregulated in OFBa. 1 *IRX9* and 1 *IRX10* were upregulated in OFBa. 2 *UXS* genes were upregulated, while 2 *UXS* genes were downregulated in OFBa compared to the control. 1 *GXM* gene was downregulated in OFBa compared to the control. 1 *GUX* gene was upregulated, while 1 *GUX* gene was downregulated in OFBa compared to the control (Fig. 6 and Table S10). The sucrose synthase (SUS) or invertase (INV)-mediated conversion pathway breaks down sucrose to yield uridine diphosphate glucose (UDP-D-Glc). CESA then utilizes UDP-D-Glc as a substrate to synthesize β-1,4 glycosidic bond-linked glucan chains, which is a crucial step for cellulose synthesis [33]. A total of 1 *SUS4* gene and 9 *INV* genes were upregulated in OFBa (Fig. 6 and Table S10).

### Characterization of key enzymes in the lignin biosynthesis pathway and the response of the transcription factor *MYB* to OFBa

**Fig. 7:**
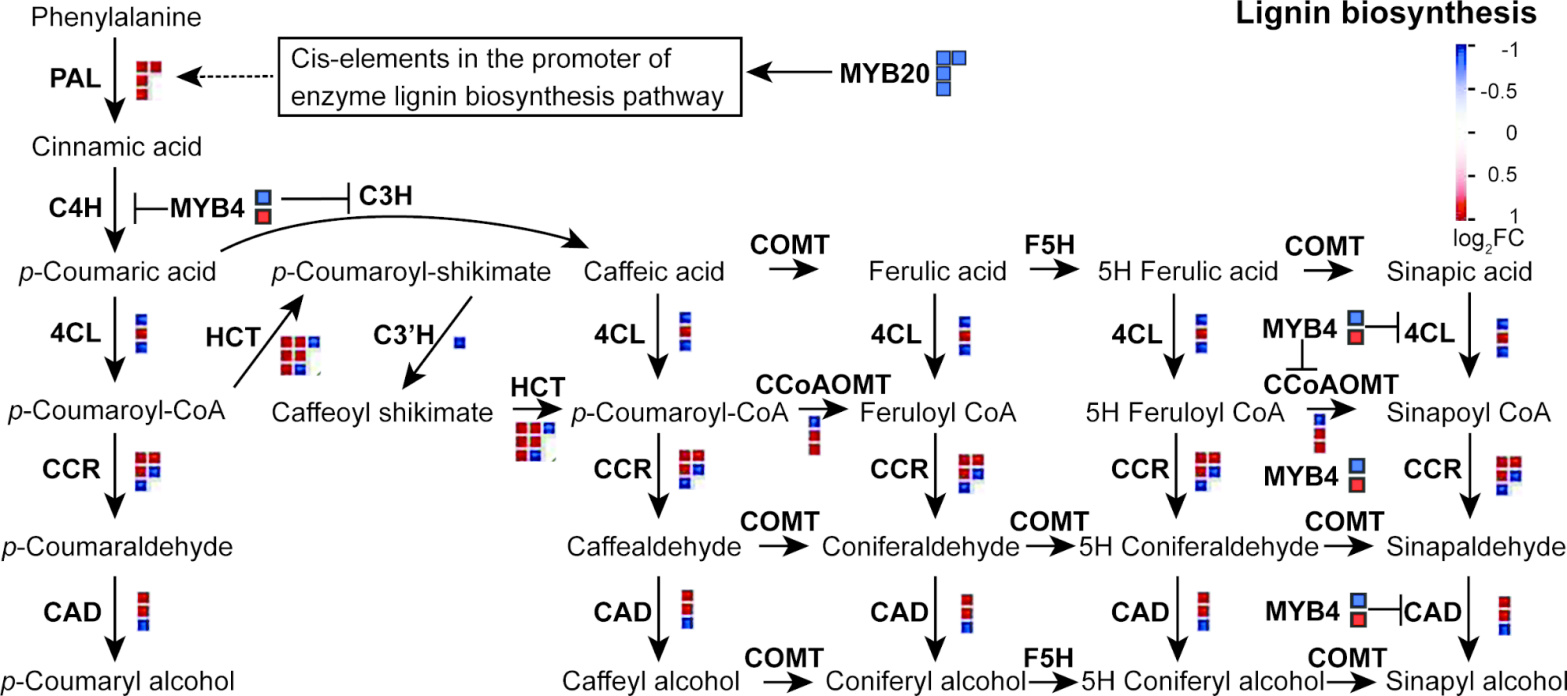
Expression patterns of lignin biosynthesis-related genes and MYB transcription factors in secondary cell walls of *Dendrocalamus farinosus* culms under OFBa. Blue and red squares represent down and upregulated DEGs, respectively. OFBa: bio-organic fertilizer (containing *Bacillus amyloliquefaciens*)

Lignin biosynthesis is a complex process that involves multiple enzymatic reactions [37]. Thus, we investigated the response mechanisms of key enzyme-related genes in the lignin synthesis pathway to OFBa treatment by mapping DEGs to this pathway. We identified 32 genes involved in the lignin biosynthesis pathway: 18 were upregulated and 14 downregulated. Among these, 4 *MYB20* transcription factors (v-myb avian myeloblastosis viral oncogene homolog 20) and 1 *MYB4* transcription factor were downregulated, while 1 *MYB4* was upregulated in OFBa compared to the control (Fig. 7 and Table S11). Under OFBa treatment, transcription levels of several genes encoding enzymes involved in the early steps of phenylpropanoid metabolism were upregulated, including 4 *PALs*, 1 *4CL*, 5 *HCTs*, 2 *CCoAOMTs*, 3 *CCRs*, and 2 *CADs*. Notably, 2 *4CLs*, 2 *HCTs*, 1 p-coumarate 3-hydroxylase (*C3H*), 1 *CCoAOMT*, 2 *CCRs*, and 1 *CAD* gene were downregulated in OFBa. This downregulation may be associated with the reduced expression of *A. thaliana* homologous transcription factors *MYB4* and *MYB20* (Fig. 7 and Table S11).

## Validation of DEGs expression in SCW biosynthesis

**Fig. 8:**
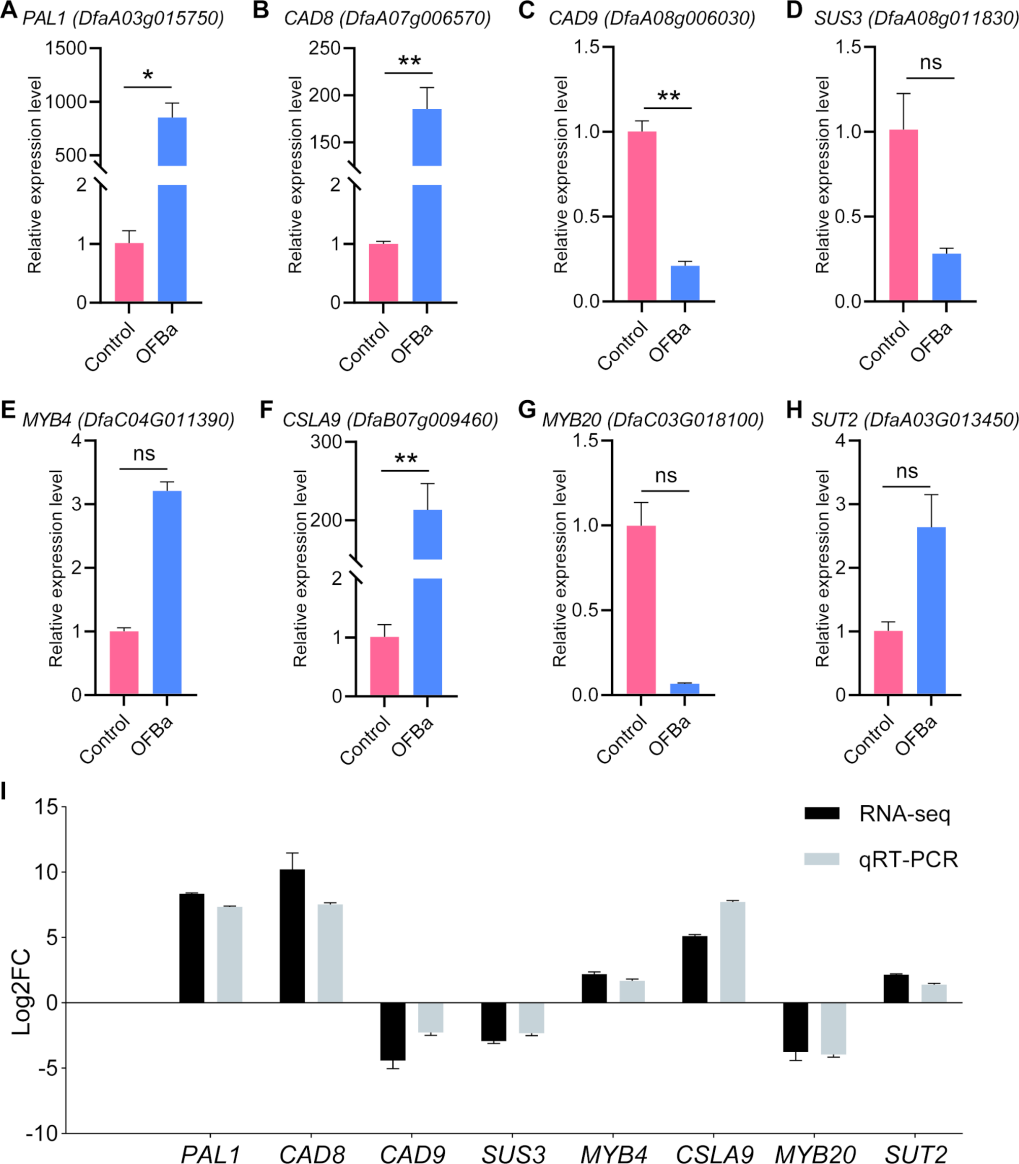
qRT-PCR validation of key genes *PAL1* (A), *CAD8* (B), *CAD9* (C), *SUS3* (D), *MYB4* (E), *CSLA9* (F), *MYB20* (G) and *SUT2* (H) for secondary wall synthesis in culms under OFBa, along with their comparative analysis against transcriptome data (I). Tukey post-hoc tests were used to calculate the differences between the control and OFBa. * indicates significant difference at 0.05 level. ** indicates significant difference at 0.01 level (*n* = 3), and ns indicates no statistical significance

We selected key structural genes and transcription factors involved in the metabolic pathway related to SCW biosynthesis in *D. farinosus* internodes under OFBa treatment for qRT-PCR analysis to validate the reliability of the transcriptome data. 8 DEGs were selected, including *DfaA03g015750* (*PAL1*), *DfaA07g006570* (*CAD8*), *DfaA08g006030* (*CAD9*), *DfaA08g011830* (*SUS3*), *DfaC04G011390* (*MYB4*), *DfaB07g009460* (*CSLA9*), *DfaC03G018100* (*MYB20*), and *DfaC08G021670* (*SUT2*) (Table S1). The results indicated that the expression levels of 5 genes—*DfaA03g015750* (*PAL1*), *DfaA07g006570* (*CAD8*), *DfaC04G011390* (*MYB4*), *DfaB07g009460* (*CSLA9*), and *DfaC08G021670* (*SUT2*)—were upregulated, while 3 genes—*DfaA08g006030* (*CAD9*), *DfaA08g011830* (*SUS3*), and *DfaC03G018100* (*MYB20*)—were downregulated (Fig. 8A-H). Further analysis of the fold change of the candidate genes revealed that their relative expression trends were consistent with the transcriptome sequencing results, confirming the accuracy of the RNA-seq data (Fig. 8I).

## Discussion

The phenylpropanoid pathway is one of the most studied, producing diverse bioactive compounds through enzymatic modifications like acylation, methylation, and glycosylation [7, 8]. Phenylpropanoid-derived polymers, such as lignin, provide mechanical support for plant growth, facilitate long-distance transport of water and nutrients, and aid in stress responses [5]. As a dual-purpose bamboo species for bamboo shoots and timber, the main components of SCW, such as lignin, hemicellulose, and cellulose, play a crucial role in the application and economic value of *D. farinosus*. In this study, we used metabolomics to identify specific changes in the phenylpropanoid metabolic pathway in bamboo shoots under bio-organic fertilizer treatment. We further examined related biosynthetic pathways and regulatory mechanisms through transcriptomic data from both bamboo shoots and bamboo culms.

Our study found that differential metabolites in bamboo shoots were significantly enriched in phenylpropanoids and derivatives under OFBa treatment (Fig. 1D). As sessile organisms, plants must adapt to changing environmental conditions. Phenylpropanoids are essential secondary metabolites involved in plant responses to biotic and abiotic stresses [38]. Studies have shown that sucrose induction leads to an increase in SnRK1-regulated anthocyanin biosynthesis [39]. Similarly, exogenous jasmonic acid and its derivatives, such as 12-hydroxyjasmonic monoacyl isoleucine, induce anthocyanin accumulation in plants like *Arabidopsis thaliana*, tomato, and sorghum [40]. Exogenous glucose activates the *MdHXK1* sensor, which phosphorylates *MdbHLH3*. This phosphorylation stabilizes *MdbHLH3* and enhances its transcriptional activation of anthocyanin biosynthesis genes, thereby promoting anthocyanin accumulation in apple [41]. In apple, exogenous growth hormone treatments induced the degradation of *MdIAA121* and released *MdARF13*, a negative regulator of anthocyanin biosynthesis [42]. Our study suggests that OFBa may act similarly as an exogenous agent, inducing gene expression in bamboo shoots and causing perturbations in the phenylpropanoid metabolic pathway (Fig. 4).

Our study found that *D. farinosus* grown under OFBa showed a significantly upregulated in the expression of genes related to sugar and starch metabolism, especially within the sugar metabolism pathway (Fig. 6). SCW biosynthesis is a complex process, involving multiple gene families directly engaged in polysaccharide biosynthesis and the remodeling of cell wall components [3]. It also includes structural proteins involved in cell wall assembly and rearrangement [43]. This process is transcriptionally regulated by specific transcription factors. Cellulose synthase (*CESA*), for example, uses UDP-α-D-Glc as a substrate for synthesizing β-1,4-linked glucan chains (Fig. 6). β-1,4-linked glucan chains are produced from sucrose, a major product of photosynthesis that supports carbon partitioning and cellulose biosynthesis [44]. This expression pattern may explain the significant increase in cellulose content observed (Fig. S4C) [19]. Meanwhile, our results indicate that nine INV-like genes are highly expressed in OFBa-treated samples and may provide more substrates for cellulose synthesis compared to SUS genes (Fig. 6 and Table S10). Sucrose synthase (SUS) converts sucrose to UDP-Glc and fructose either directly or through an invertase (INV)-mediated pathway. While earlier studies suggested that SUS-mediated UDP-Glc contributes to cellulose biosynthesis in cotton fibers [45], genetic analyses in *A. thaliana* and *Populus spp.* indicated that SUS gene mutations or downregulation had minimal effects on cellulose synthesis and plant growth [46, 47]. This suggests that sucrose synthase is unlikely to play a major role in providing UDP-Glc for cellulose synthesis. This supports the hypothesis that the invertase-mediated pathway is the primary source of UDP-Glc for cellulose biosynthesis. Existing evidence shows that simultaneous mutations in two *A. thaliana* cytoplasmic invertase genes (*CINV*) lead to severe growth defects [46]. Further biochemical and imaging analyses revealed insufficient production of UDP-Glc and cellulose, as well as altered cellulose microfibril organization [48]. Downregulation of the wood-related *CINV* gene in poplar also reduced crystalline cellulose and UDP-Glc levels in transgenic *Populus spp.* [47].

*MYB20* is a transcription factor that directly activates lignin biosynthesis genes and phenylalanine biosynthesis genes during secondary wall formation in *A. thaliana* [14]. In our study, all four identified *MYB20* genes were significantly downregulated in OFBa-treated samples (Fig. 7 and Table S11). It has been studied that the absence of *MYB20* leads to growth and developmental defects, along with a significant reduction in lignin biosynthesis. Moreover, *MYB20/42/43/85* has been shown to directly activate transcriptional repressors *MYB4/7/32* which specifically inhibit chalcone synthase (CHS) expression, an enzyme catalyzing the first step in flavonoid biosynthesis [14, 49]. Our results showed that a *D. farinosus MYB4* exhibited a significant upregulated of expression levels under OFBa (Log2FC = 2.32) (Fig. 7 and Table S11). As CHS also requires phenylalanine as a precursor, it competes with lignin biosynthesis [50]. Thus, it remains to be investigated whether MYB20 from D. farinosus directly binds to *MYB4* under OFBa treatment, resulting in a redistribution of carbon flux within the phenylpropanoid metabolic pathway [51] and, subsequently, a decrease in lignin content (Fig. S4B). Transient expression analysis in *Nicotiana benthamiana* leaves demonstrated that *MYB20* activates the *MYB4* promoter. Zhao et al. used EMSA to investigate whether *MYB4* is a direct target [52]. The results showed that all four MBP-MYB proteins bound to the *MYB4* promoter but not to the MBP tag.

Although our results suggest that OFBa may influence the biosynthesis of bamboo culm SCW by inhibiting the phenylpropanoid metabolic pathway in bamboo shoots, the detailed molecular regulatory mechanisms are still unclear. Investigating how OFBa interacts with bamboo in the complex inter-root environment to enhance the growth of *D. farinosus* through multi-omics approaches would be a compelling topic for future research.

## Conclusion

Our results indicate that *D. farinosus* shoots grown under OFBa showed a significantly change in the phenylpropanoid pathway, while the differentially expressed genes in this pathway were notably downregulated. Meanwhile, transcriptome analysis of the stalks revealed that genes related to secondary cell wall biosynthesis—specifically those involved in cellulose, hemicellulose, and lignin biosynthesis—were significantly affected by OFBa. Additionally, we propose a model suggesting that OFBa may influence secondary cell wall biosynthesis in bamboo culms by perturbing the phenylpropanoid metabolic pathway in bamboo shoots. The findings of this study could offer new insights and approaches for the efficient utilization of bamboo and wood fiber biomass.

## Materials and methods

### Plants and fertilizers

Potted *D. farinosus* were planted at the experimental base of the College of Life Science and Engineering, Southwest University of Science and Technology, Mianyang, Sichuan, China (geographic location: 31°32’44 “N, 104°41’402 “E, altitude: 480 m). Topsoil (0–20 cm depth) was collected from the test site and screened to remove rocks and plant litter. The soil contained 37% salt, 36% clay and 27% sand. The physico-chemical properties of the soil were as follows: pH 6.49 ± 0.01, conductivity 112.93 ± 1.06 µS cm^− 1^, total carbon 1220 ± 102 mg kg^− 1^, total organic carbon 511 ± 12 mg kg^− 1^, total nitrogen 353 ± 11 mg kg^− 1^, total phosphorus 180 ± 9 mg kg^− 1^, available nitrogen 142 ± 11 mg kg^− 1^ and available phosphorus 22.4 ± 7.3 mg kg^− 1^ [20].

Two types of fertilizers were used in this study. Organic fertilizer (organic matter ≥ 45%, N + P2O5 + K2O ≥ 5%, N [1.4%], P [4.5%] and K [1.7%]) was purchased from Lotus Environmental Technology Fertilizer Company (Henan, China). *Bacillus amyloliquefaciens* microbial fertilizer (microbial, 100 billion CFU /g) was purchased from Nongbao; *B. amyloliquefaciens* is a typical plant-growth-promoting rhizobacterium (PGPR) that promotes plant growth and improves nitrogen use efficiency.

### Sample collection and preparation

Fertilizer was first added to the collected topsoil. Each plastic pot was then filled with 50 kg of the soil-fertilizer mixture: 44 cm diameter at the top, 28 cm diameter at the bottom and 30 cm high. The treatments were as follows: (1) Control: no fertilizer; and (2) OFBa treatment: 500 g of organic fertilizer with an additional 30 g of *B. amyloliquefaciens* (equivalent to 0.4–0.8 g per kg of potting soil) [20]. Three biological replicates were performed for each treatment condition. Healthy potted bamboo plants, defined as having no visible signs of disease, pest infestation, or physiological stress and of similar size, were transplanted into pots containing same soils and watered. The plants were then watered weekly during the growing season to allow the water to pass through the potting soil. All *D. farinosus* used in the experiment were annual bamboos. After the start of the bamboo shoot season, young shoots samples were collected at 15 days post-growth for the transcriptome and metabolome analyses. Bamboo samples were collected after twigging and leaf spreading (about 90 d of growth) for transcriptome sequencing. Freshly collected samples were quickly frozen in liquid nitrogen and stored at − 80℃ until the total RNA and metabolite extractions.

### Hand-sectioning and phloroglucinol staining for lignin detection

Thin cross-Sects. (50–100 μm thick) of fresh plant internodes containing secondary cell wall (SCW) structures were carefully prepared using a sharp scalpel or razor blade. For staining, a phloroglucinol solution (0.5–1% w/v in 95% ethanol) was freshly prepared and mixed with hydrochloric acid at a 1:1 volume ratio. The sections were immersed in the phloroglucinol solution for 2–5 min, followed by the addition of a few drops of concentrated hydrochloric acid (HCl) to enhance staining, which continued for an additional 30 s to 1 min. After gently rinsing with water, the sections were placed on slides, with a drop of hydrochloric acid added, and covered with a coverslip. Under microscopic observation, lignified areas stained red or purplish-red, highlighting xylem and other lignin-containing cells [28].

### Liquid chromatography/mass spectrometry non-targeted metabolomics (LCMS) analysis

An accurately weighed sample (80 mg) was transferred into a 1.5 ml Eppendorf tube. The control and fertilized treatments were performed in three biological replicates. Metabolites in bamboo shoots were extracted with methanol and water (vol: vol = 7:3). After filtration of the supernatant through a 0.22-µm filter, the supernatant was transferred to an LC injection vial and stored at -80 °C until LC-MS analysis. A Nexera UPLC system (Shimadzu Corporation, Japan) coupled with Q-Exactive quadrupole-Orbitrap mass spectrometer equipped with heated electrospray ionization (ESI) source (Thermo Fisher Scientific, Waltham, MA, USA) was used to analyze the metabolic profiling in both ESI positive and ESI negative ion modes. An ACQUITY UPLC HSS T3 column (1.8 μm, 2.1 × 100 mm) were employed in both positive and negative modes. The binary gradient elution system consisted of (A) water (containing 0.1% formic acid, v/v) and (B) acetonitrile (containing 0.1% formic acid, v/v) and separation was achieved using the following gradient: 0 min, 5% B; 2 min, 5% B; 4 min, 25% B; 8 min, 50% B; 10 min, 80% B; 14 min, 100% B; 15 min, 100% B; 15.1 min, 5% and 16 min, 5% B. The flow rate was 0.35 mL/min and column temperature was 45 ℃. All the samples were kept at 4℃ during the analysis. The injection volume was 2 µL. Compound identification were based on precise mass-to-charge ratio (M/z), secondary fragments, and isotopic distribution using the Human Metabolome Database (HMDB), Lipidmaps (V2.3), Metlin, and self-built databases to do qualitative analysis [29].

Principal Component Analysis (PCA) was used to observe the overall distribution among the samples and the stability of the whole analysis process. Orthogonal Partial Least-Squares-Discriminant Analysis (OPLS-DA) was utilized to distinguish the metabolites that differ between groups. To prevent overfitting, 7-fold cross-validation and 200 Response Permutation Testing (RPT) were used to evaluate the quality of the model. Variable Importance of Projection (VIP) values obtained from the OPLS-DA model were used to rank the overall contribution of each variable to group discrimination. A two-tailed Student’s T-test was further used to verify whether the metabolites of difference between groups were significant. Differential metabolites were selected with VIP values greater than 1.0 and p-values less than 0.05.

### Transcriptome sequence analysis

The samples for RNA sequencing were 15-day-old bamboo shoots and 1, 7, and 14 internodes of 90-day-old adult bamboos. The control and fertilized treatments were performed in three biological replicates. Total RNA was extracted using TRIzol reagent (Tiangen, China), and any potential genomic DNA contamination was removed by DNase treatment. Ribosomal RNA was depleted using the Ribo-Zero Gold Kit (Illumina, Beijing, China). The quantity and quality of total RNA was checked using NanoDrop 2000 spectrophotometer (Thermo Fisher Scientific, Beijing, China), Qubit 2.0 and Agilent 2100. Qualified RNAs were used to construct library using the poly(A) enrichment protocol [53]. All samples were processed using the HiSeq 2500 platform (Illumina, Beijing, China), enerating paired-end reads of 150 bp. Reference genome was pre-defined for the analysis (https://ngdc.cncb.ac.cn/gwh, GWHFHFY00000000.1). Each sample produced approximately 20–30 million reads [54]. Quality control of raw reads was conducted using FastQC [55], ensuring the removal of low-quality reads and adapter sequences. Cleaned reads were mapped to the reference bamboo genome using HISAT2 [56], and mapped reads were quantified with HTSeq [57]. The analysis design for differentially expressed genes (DEGs) were Control: no fertilization versus OFBa: 500 g of organic fertilizer and 30 g of *B. amyloliquefaciens*. For experiments with biological replicates, differential expression analysis is processed by DESeq2 [30]. Criteria for differentially expressed genes was set as FC ≥ 2 and false discovery rate (FDR) < 0.01. Functional annotation of DEGs and enrichment analysis were performed using the following databases: Nr (NCBI non-redundant protein sequences); Nt (NCBI non-redundant nucleotide sequences); Pfam (Protein family); KOG/COG (Clusters of Orthologous Groups of proteins); Swiss-Prot (A manually annotated and reviewed protein sequence database); KO (Kyoto Encyclopedia of Genes and Genomes, KEGG Ortholog database); GO (Gene Ontology). We used KOBAS (http://bioinfo.org/kobas) software to test the statistical enrichment of DEGs in KEGG (http://www.genome.jp/kegg/) pathways. A visual analysis of the metabolic pathways of the DEGs (such as cellular response overview, photosynthetic metabolism and mitochondrial electron transport chain) was performed using Mapman software (version 3.6.0 RC1) [31].

The Metabolomics and transcriptome sequencing were conducted by OE Biotech Co., Ltd. (Shanghai, China) and Biomarker Technologies Co, Ltd. (Beijing, China), respectively.

### Real-time PCR analysis

Total RNA was extracted with Trizol reagent (Invitgen) and treated with ribonuclease-free DNase I (Takara) to eliminate DNA contamination. The concentration and purity of RNA were measured using a NanoDrop 1000 spectrophotometer from TermoFisher Science. RNA integrity was tested using a BioAnalyzer 2100 (Agilent). 2 µg of total RNA was synthesized using the Superscript III First Strand Synthesis Kit (Invitgen) with oligodeoxyribonucleic acid (Olio DT) primers according to the manufacturer’s instructions. Real-time quantitative PCR (qRT) was carried out using the LightCycler^®^ 480 system (Roche) and SYBR PreMix Ex Taq II (Takara). -PCR) analysis. Expression normalization was performed using *Tubulin* (the genes encoding microtubule proteins) as the internal reference gene [13, 32]. The primer details are listed in Table S1. Expression of relevant genes was calculated by the 2^−ΔΔCt^ method (R. Hu et al., 2017).

### Statistical analysis

GraphPad Prism 8.0.1 (Microsoft Window, United States) software was used to generate bar graphs and box plots. Statistical analysis was performed using SPSS Statistics 22.0 software (IBM, United States). Statistical plots (e.g., heat maps, principal component analysis (PCA), Venn diagram) used in this study were drawn using the OmicShare online analysis platform. Image layout was performed using Adobe Illustrator CS6 (Adobe, United States).

## Electronic supplementary material

Below is the link to the electronic supplementary material.

Supplementary Material 1

Supplementary Material 2

Supplementary Material 3

Supplementary Material 4

Supplementary Material 5

Supplementary Material 6

Supplementary Material 7

Supplementary Material 8

## Data Availability

The whole genome sequence data reported in this paper have been deposited in the Genome Warehouse in National Genomics Data Center [58], Beijing Institute of Genomics, Chinese Academy of Sciences/China National Center for Bioinformation [59], under accession number GWHFHFY00000000.1 that is publicly accessible at https://ngdc.cncb.ac.cn/gwh. The RNA-seq data which supports the findings of this study are linked in https://www.ncbi.nlm.nih.gov/, PRJNA1184376.
