# Supplementary material for: Bio-organic fertilizer affects secondary cell wall biosynthesis of Dendrocalamus farinosus by inhibiting the phenylpropanoid metabolic pathway
Source: BMC Plant Biol. 2024 Nov 22;24:1112. doi: 10.1186/s12870-024-05825-8 (PMC11583417; doi:10.1186/s12870-024-05825-8)
Supplement: Supplementary file 1 — Supplementary Material 1 [file 12870_2024_5825_MOESM1_ESM.docx]

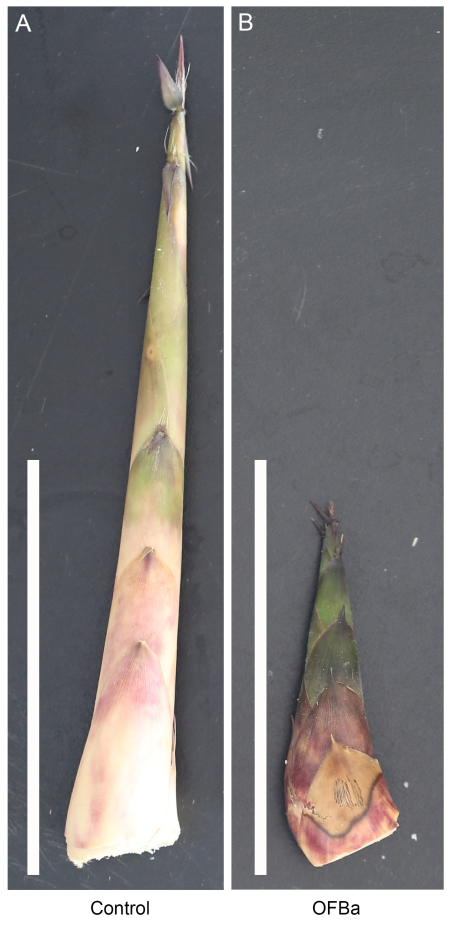


Figure S1 Phenotypes of bamboo shoots used for metabolome and transcriptome. The control (A) and OFBa (B). Scale bar, 10 cm. OFBa: bio-organic fertilizer (containing *Bacillus amyloliquefaciens*).


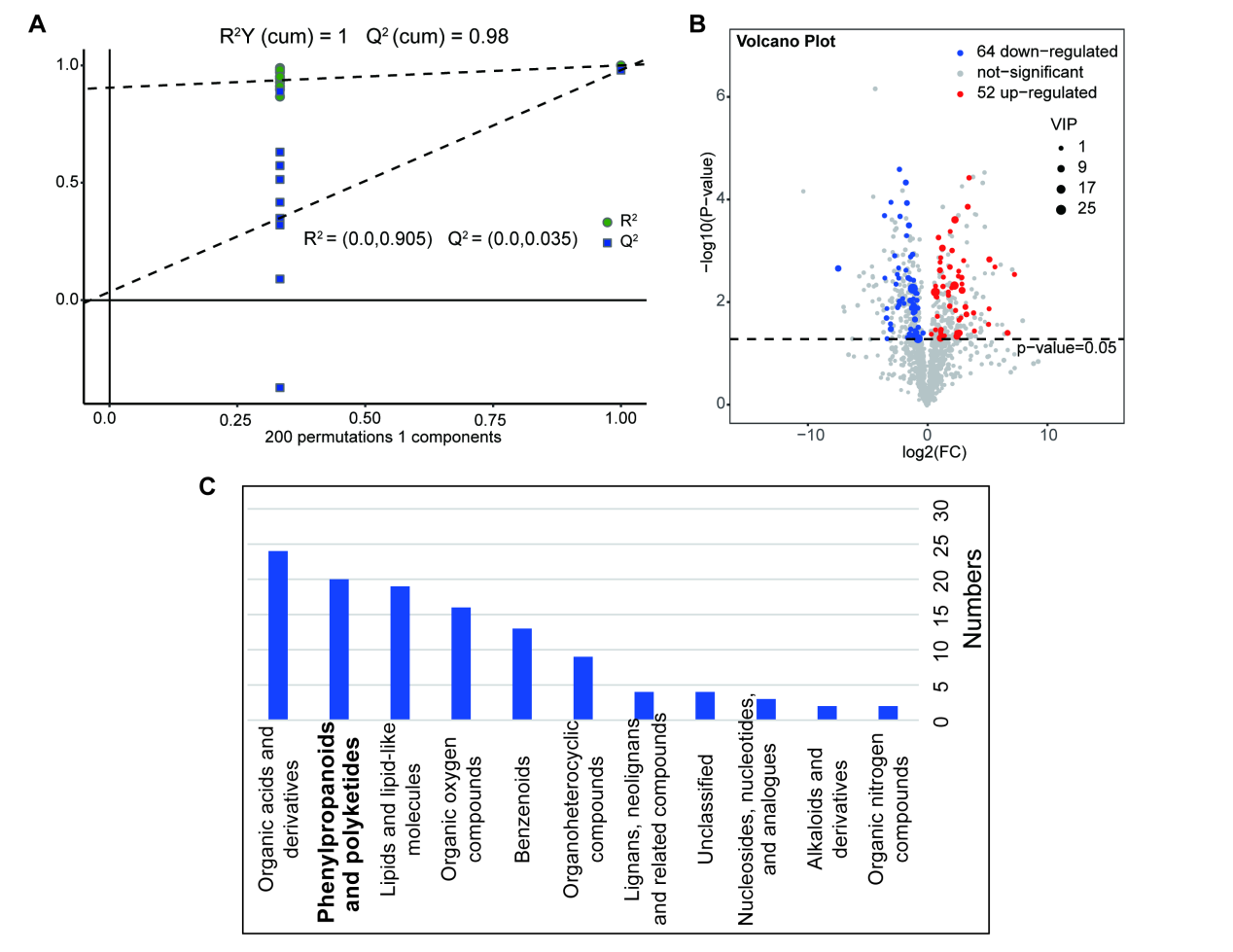


Figure S2 Metabolism profile of *Dendrocalamus farinosus* shoots under OFBa treatment. Orthogonal Partial Least Squares Discriminant Analysis (OPLS-DA) (A), volcano plot of differentially expressed metabolites (B) and metabolic pathway enrichment maps for top20 (C).

OFBa: bio-organic fertilizer (containing *Bacillus amyloliquefaciens*).


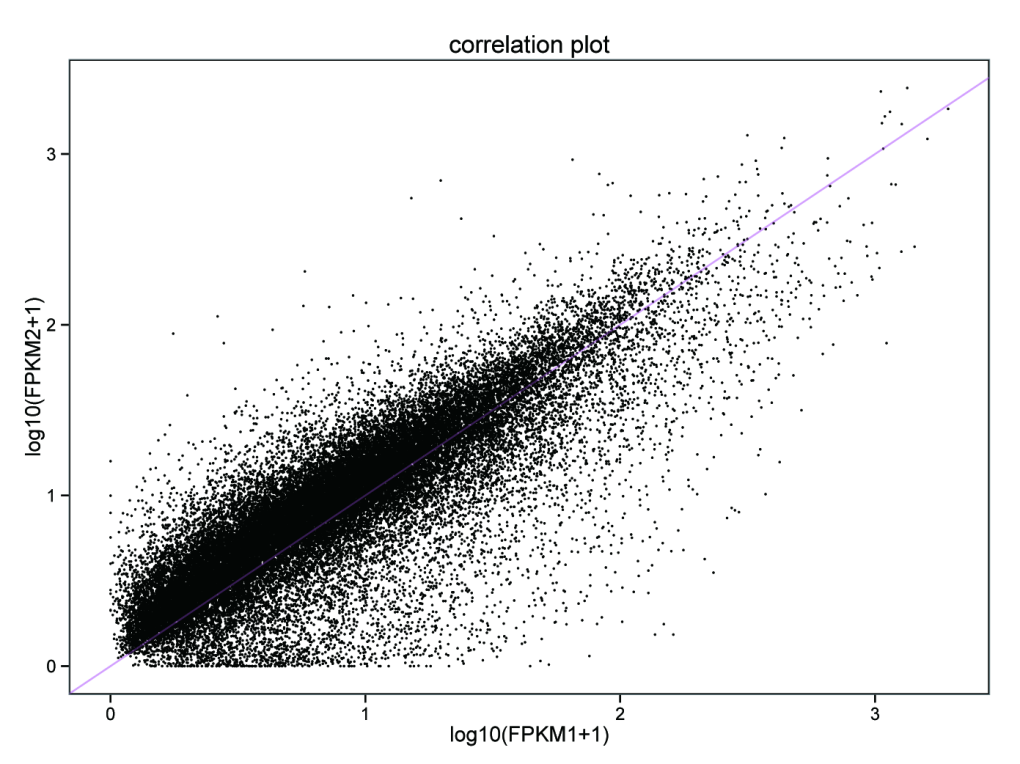


Figure S3 Correlation plot between the samples (FPKM values) of *Dendrocalamus farinosus* shoots under OFBa . OFBa: bio-organic fertilizer (containing *Bacillus amyloliquefaciens*).


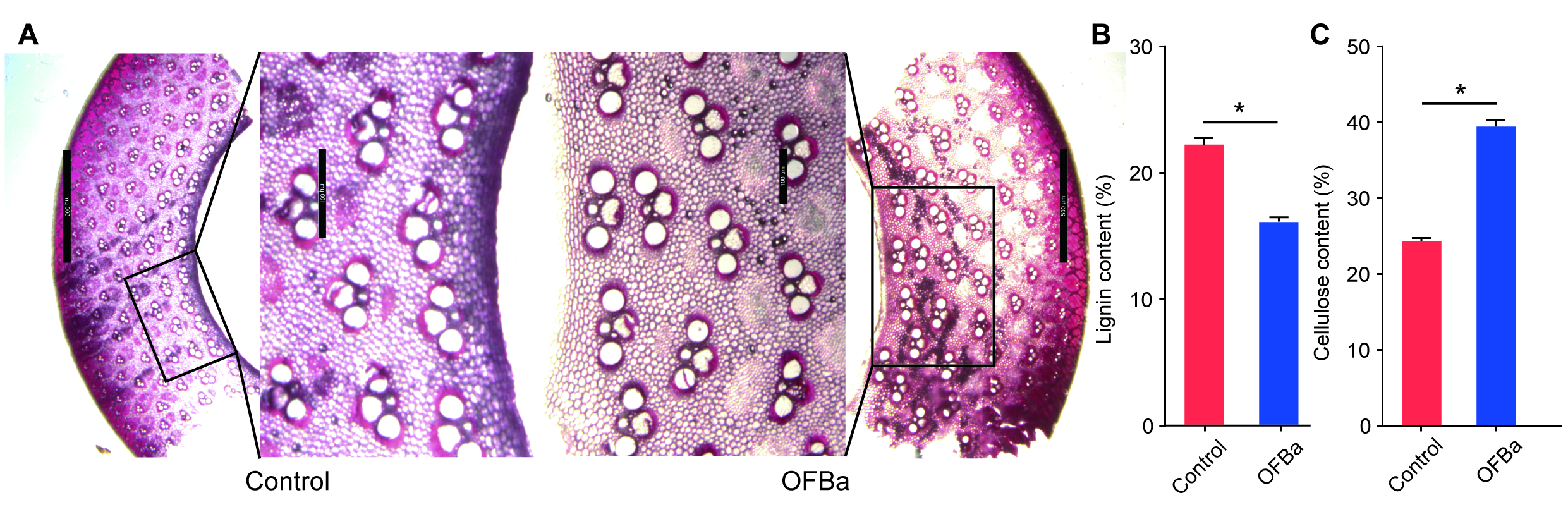


Figure S4 Phloroglucinol staining (A), lignin content (B) and cellulose content (C) of *Dendrocalamus farinosus* culms under OFBa.

Note: In Fig. S4A, the scale within the black box represents 100 μm, while the scale outside the black box represents 500 μm. OFBa: bio-organic fertilizer (containing *Bacillus amyloliquefaciens*). Tukey post-hoc tests were used to calculate the differences between the control and OFBa. * indicates significant difference at 0.05 level (n=3). OFBa: bio-organic fertilizer (containing *Bacillus amyloliquefaciens*).


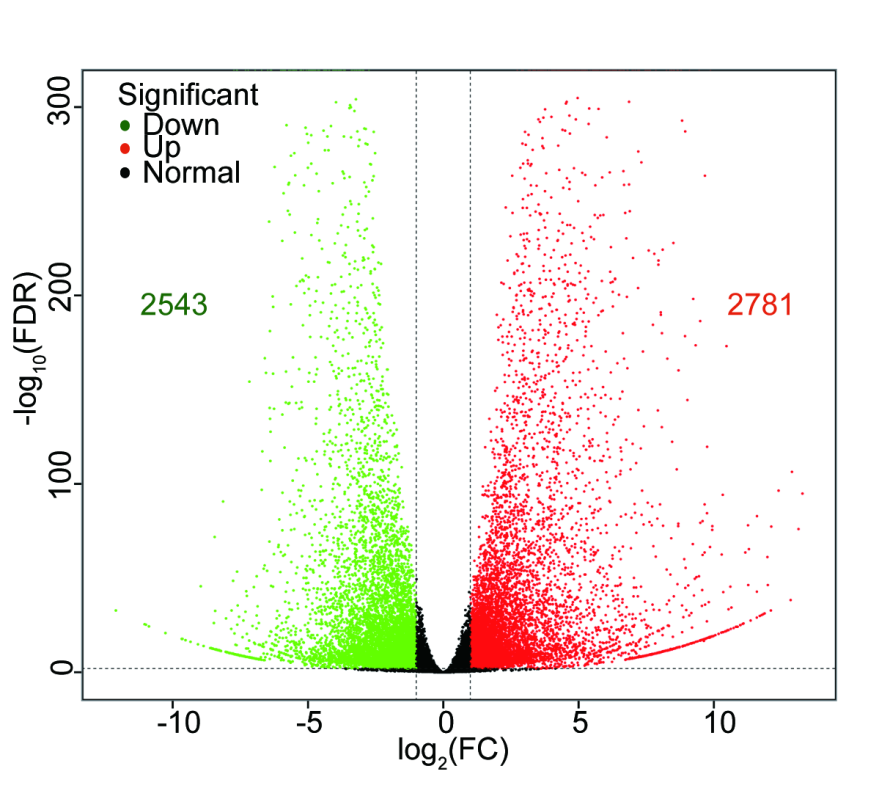


Figure S5 Volcano plot of 5324 differentially expressed genes in the culm transcriptome. All of the differentially expressed genes were common to all three internodes under OFBa compared with the control. OFBa: bio-organic fertilizer (containing *Bacillus amyloliquefaciens*).

Table S1 Specific primers of candidate genes for qRT-PCR

| Gene_ID | Swiss-Prot annotation | Forward primer (5'-3') | Reverse primer (5'-3') |
| --- | --- | --- | --- |
| DfaA03g015750 | PAL1 | ATCAACACTCTCCTTCAAG | TGTGACATTGGCATTCAG |
| DfaA07g006570 | CAD8 | GGCACTGGAGATGATGAC | CATTCTTGATGTTGTGTAGGT |
| DfaA08g006030 | CAD9 | AACAAATGAAGGCTGCTAT | ATGATGAGTGGTGTCAGA |
| DfaA08g011830 | SUS3 | ATGATACATCTCCTTCTG | ATACTACCACAACATTGA |
| DfaC04G011390 | MYB4 | CTCGGAGGATTTCCAGAT | TCCACTGAGACATCGTAG |
| DfaB07g009460 | CSLA9 | GAAGAGGCGAACAAGATT | CGTAGCAACCACATAGAAT |
| DfaC03G018100 | MYB20 | GAGAACGGACAACGAGAT | CTGGTGAGTGATGGGATC |
| DfaC08G021670 | SUT2 | CCTCTCGCCATGGACTCC | GGCCTCCTCTCCACATGG |
| *Tubulin* | / | GCCGTGAATCTCATCCCCTT | GCCGTGAATCTCATCCCCTT |

*Tubulin* was used as an internal reference gene.

Table S7 Statistics of differentially expressed genes related to phenylpropanoid metabolic pathway that are homologous to *Arabidopsis* in the shoots

| Gene id | Annotation  *Arabidopsis thaliana*  (Mouse-ear cress) | Log_2_FC |
| --- | --- | --- |
| Dfaa03g015730 | *PAL* | -3.3663483 |
| Dfaa03g015750 | *PAL* | -3.0866842 |
| Dfaa04g012110 | *PAL* | -5.0609894 |
| Dfab03g015230 | *PAL* | -2.0271773 |
| Dfab04g014650 | *PAL* | -1.8197753 |
| Dfab04g014660 | *PAL* | -4.362742 |
| Dfac03g013900 | *PAL* | -3.1118202 |
| Dendrocalamus_farinosus_  newgene_32659 | *PAL* | -2.9189312 |
| Dendrocalamus_farinosus_  newgene_13416 | *PAL* | -3.4395232 |
| Dfaa03g009850 | *C4H* | -2.5749607 |
| Dfaa03g009850 | *C4H* | -1.9588003 |
| Dfab03g009470 | *C4H* | -2.2351341 |
| Dfac01g012310 | *C4H* | -1.9349551 |
| Dfaa03g003070 | *4CL* | -1.6433053 |
| Dfaa07g012650 | *4CL* | -5.624884 |
| Dfab03g003100 | *4CL* | -3.7155488 |
| Dfac03g007240 | *4CL* | -1.8906926 |
| Dfac03g016490 | *4CL* | -3.0247226 |
| Dfac08g006630 | *4CL* | -3.0256016 |
| Dfac10g002130 | *4CL* | 2.180384 |
| Dfa0g018570 | *4CL* | -3.14472 |
| Dfaa07g003120 | *COMT* | -3.3944137 |
| Dfab06g011840 | *COMT* | -1.912288 |
| Dfaa03g014800 | *HCT* | -4.2746396 |
| Dfab03g014540 | *HCT* | -2.2275224 |
| Dfac03g013140 | *HCT* | -2.6435945 |
| Dfac06g009170 | *HCT* | 1.6108471 |
| Dfac11g006250 | *HCT* | -4.409827 |
| Dfaa01g008230 | *C3H* | 2.2692847 |
| Dfaa07g014340 | *CCoCOMT* | -3.0049927 |
| Dfab07g011900 | *CCoCOMT* | -2.0140965 |

| Gene id | Annotation  *Arabidopsis thaliana*  (Mouse-ear cress) | Log_2_FC |
| --- | --- | --- |
| Dfab11g015250 | *CCoCOMT* | -1.8720722 |
| Dfaa03g003390 | *CCR* | -1.8655438 |
| Dfaa05g001030 | *CCR* | -2.1380224 |
| Dfaa09g000340 | *CCR* | -2.9779274 |
| Dfab01g006530 | *CCR* | 2.4245975 |
| Dfab02g001040 | *CCR* | -2.6982346 |
| Dfab03g003320 | *CCR* | -5.4395127 |
| Dfab03g023830 | *CCR* | -4.7477236 |
| Dfaa11g004270 | *CAD* | -3.5750825 |
| Dfab07g004070 | *CAD* | -5.2743096 |
| Dfaa10g007280 | *CHS* | -1.950831 |
| Dfaa10g007300 | *CHS* | -1.7977284 |
| Dfaa08g026430 | *CHI* | -3.9886596 |
| Dfaa01g028150 | *FLS* | -3.1014314 |
| Dfaa01g028160 | *FLS* | -1.8905001 |
| Dfaa08g005580 | *FLS* | -4.8399787 |
| Dfaa08g027990 | *FLS* | -2.146247 |
| Dfab04g003090 | *FLS* | -3.0374386 |
| Dfab04g016520 | *FLS* | -1.6524868 |
| Dfab09g000840 | *FLS* | -1.7988552 |
| Dfab09g000880 | *FLS* | -1.5615692 |
| Dfa0g075420 | *FLS* | -2.0186374 |
| Dendrocalamus_farinosus_  newgene_19484 | *FLS* | -1.6848018 |
| Dfac03g019470 | *FLS* | -5.188746 |
| Dfaa08g005580 | *FLS* | -4.8399787 |
| Dfac01g019480 | *FLS* | -3.8737173 |
| Dfaa08g017350 | *F3’H* | 3.8316138 |
| Dfab06g006020 | *F3’H* | -2.0184057 |
| Dfab07g014240 | *F3’H* | -4.2959404 |
| Dfaa11g003550 | *DFR* | -1.7197686 |
| Dfac08g002880 | *DFR* | -1.9236804 |
| Dfaa08g007760 | *LAR* | 1.9378121 |
| Dfab01g008120 | *UFGT* | -1.8516932 |
| Dfaa11g003550 | *ANR* | -1.7197686 |
| Dfac08g002880 | *ANR* | -1.9236804 |

Fold change (FC) refers to the ratio of gene expression in two samples.

Table S10 Statistics of differentially expressed genes related to cellulose and hemicellulose biosynthesis that are homologous to *Arabidopsis* in the culm

| Gene id | Annotation  *Arabidopsis thaliana*  (Mouse-ear cress) | Log_2_FC |
| --- | --- | --- |
| DfaC02g012640 | *CESA1* | 3.762169 |
| DfaB05g006070 | *CESA2* | 2.8451676 |
| DfaC05g011700 | *CESA2* | 2.0167708 |
| Dfa0g005560 | *CESA4* | 2.6765475 |
| DfaC08g016650 | *CSLA02* | -2.3030024 |
| DfaC08g016650 | *CSLA02* | -2.3030024 |
| DfaA07g012100 | *CSLA09* | 1.4097153 |
| DfaA11g012940 | *CSLA09* | -3.7141402 |
| DfaB07g009460 | *CSLA09* | 5.2317915 |
| Dfa0g017510 | *CSLA09* | -3.9042494 |
| DfaA07g012100 | *CSLA09* | 1.4097153 |
| DfaA11g012940 | *CSLA09* | -3.7141402 |
| DfaB07g009460 | *CSLA09* | 5.2317915 |
| Dfa0g017510 | *CSLA09* | -3.9042494 |
| DfaB04g010400 | *CSLB04* | -2.9664986 |
| DfaB04g010410 | *CSLB04* | -1.1819221 |
| DfaA02g003860 | *CSLC12* | -3.3152084 |
| DfaB08g024240 | *CSLC12* | -5.6368423 |
| DfaC11g017260 | *CSLC12* | -5.705036 |
| DfaC11g009080 | *CSLD2* | -5.4801145 |
| Dfa0g033400 | *CSLD2* | -5.5228763 |
| DfaB05g011240 | *CSLD3* | 4.871713 |
| DfaA06g007120 | *CSLE1* | -2.5619175 |
| Newgene_2605 | *CSLE1* | -2.2571084 |
| DfaB01g000440 | CSLG2 | -3.1203558 |
| DfaA05g013550 | COBL4 | -2.9154427 |
| DfaC05g003790 | COBL4 | -4.567478 |
| DfaA08g023350 | COB | 1.4934415 |
| DfaB07g000570 | IRX7 | -2.7794235 |
| DfaB01g013390 | IRX9 | 1.2663966 |
| DfaC08g012780 | IRX9 | -3.0560871 |

| Gene id | Annotation  *Arabidopsis thaliana*  (Mouse-ear cress) | Log_2_FC |
| --- | --- | --- |
| DfaC01g005400 | *IRX10* | 2.1272235 |
| DfaB01g002250 | *IRX10* | -2.1157375 |
| DfaB02G009990 | *UXS* | -1.240758361 |
| DfaA02G009640 | *UXS* | -1.926703043 |
| Dfa0G052410 | *UXS* | 2.032446058 |
| DfaA05G016470 | *UXS* | 1.134508341 |
| DfaA10G008060 | *GXM* | -2.69041987 |
| DfaB08G004880 | *GUX* | 1.8990485 |
| DfaA03G012330 | *GUX* | -3.343699798 |
| DfaB11G014310 | *SUS4* | 1.548472778 |
| DfaA04G007080 | *CWINV2* | 7.558125068 |
| DfaB03G011430 | *CWINV2* | 4.145821576 |
| DfaC04G006630 | *CWINV2* | 6.97664293 |
| NewGene_14772 | *INVA* | 3.224858236 |
| Newgene_2842 | *INVA* | 3.0582173 |
| DfaB08G011530 | *INVA* | 3.622789495 |
| Dfa0G033130 | *CINV2* | 1.505941212 |
| DfaC03G002650 | *CINV2* | 1.642966568 |
| DfaB12G002560 | *CINV2* | 4.196153438 |

Fold change (FC) refers to the ratio of gene expression in two samples.

Table S11 Statistics of differentially expressed genes related to lignin biosynthesis that are homologous to *Arabidopsis* in the culm

| Gene id | Annotation  *Arabidopsis thaliana*  (Mouse-ear cress) | Log_2_FC |
| --- | --- | --- |
| DfaA03G020680 | *MYB20* | -4.328263023 |
| DfaB03G020240 | *MYB20* | -3.485252424 |
| DfaC03G018100 | *MYB20* | -3.50252467 |
| DfaB11G011960 | *MYB20* | -5.115162815 |
| DfaC04G011390 | *MYB4* | 2.322429458 |
| DfaA10G008830 | *MYB4* | -1.180525938 |
| DfaA03G015750 | *PAL1* | 8.432707286 |
| DfaA04G012110 | *PAL1* | 8.798784921 |
| Dfa0G072870 | *PAL1* | 8.502901509 |
| Newgene_32659 | *PAL1* | 4.968382121 |
| DfaC01G004020 | *4CL* | 4.303913143 |
| DfaA03G019070 | *4CL* | -3.886263771 |
| DfaC10G002120 | *4CL* | -1.848814813 |
| DfaA06G010320 | *HCT* | 2.956275689 |
| DfaA09G006750 | *HCT* | 8.626474958 |
| DfaA09G006770 | *HCT* | 4.15701056 |
| DfaB03G014540 | *HCT* | 1.342029477 |
| DfaB06G010120 | *HCT* | 1.481618537 |
| DfaC04G010630 | *HCT* | -3.625574503 |
| Dfa0G066090 | *HCT* | -2.738876125 |
| DfaA01G008230 | *C3H* | -1.336713232 |
| DfaA07G014350 | *CCoAOMT* | 4.529640884 |
| DfaA07G014340 | *CCoAOMT* | 2.942010098 |
| DfaA06G007370 | *CCoAOMT* | -1.839926724 |
| DfaA02G003450 | *CCR* | 3.281677656 |
| DfaA03G003390 | *CCR* | 2.218275913 |
| DfaB03G003320 | *CCR* | 4.21973464 |
| DfaB01G006530 | *CCR* | -2.983311596 |
| DfaC11G000490 | *CCR* | -4.9346614 |
| DfaA07G006370 | *CAD* | 7.187058195 |
| DfaA07G006570 | *CAD* | 9.747512023 |
| DfaA08G006030 | *CAD* | -4.120381946 |

Fold change (FC) refers to the ratio of gene expression in two samples.

Table S2 Detailed annotation of differentially expressed metabolites in bamboo shoots treated with OFBa.

Table S3 Detailed data for all soil metabolites analyzed.

Table S4 Enrichment analysis of metabolic pathways for differential metabolites in bamboo shoots under OFBa treatment.

Table S5 Statistics of differentially expressed genes in the transcriptome of bamboo shoots treated with OFBa.

Table S6 Statistics of differentially expressed genes involved in the visualization of the overall cellular response in bamboo shoots under OFBa treatment.

Table S8 Genes identified and annotated through transcriptome analysis in culms treated with OFBa.
Table S9 Differentially expressed genes in intersnode 1,7 and 14 and their shared differentially expressed genes under OFBa.
